# Efficacy of canakinumab in biologic-naïve versus previously biologic-exposed SJIA patients: a 12 week pooled post-hoc analysis

**DOI:** 10.1186/1546-0096-12-S1-P66

**Published:** 2014-09-17

**Authors:** P Quartier, A Grom, N Ruperto, HI Brunner, K Schikler, M Erguven, L Goffin, M Hofer, T Kallinich, K Marzan, C Gaillez, K Lheritier, K Abrams, A Martini, DJ Lovell

**Affiliations:** 1 Necker-Enfant Malades Hospital, Paris, France; 2 PRCSG, Cincinnati, OH, USA; 3 PRINTO-Istituto Gaslini , Genova, Italy; 4 Novartis Pharma, Basel, Switzerland; 5 Novartis Pharmaceutical Corporation, NJ, USA

## Introduction

Canakinumab (CAN), a selective, human anti-IL-1β monoclonal antibody is approved for SJIA in over 30 countries. Efficacy and safety of CAN over 12 weeks have been demonstrated in 2 phase III trials [1]. Out of these trials >60% of the pts received a previous biologic and were switched to CAN due to lack of efficacy or for safety reasons, and may be more refractory to another biologic therapy.

## Objectives

To present a post-hoc evaluation of CAN efficacy in biologic-naïve (BN) pts and those previously exposed to biologics (BE) during the first 12-weeks.

## Methods

Pooled data from CAN naïve pts, enrolled in two phase III trials^1^ and an extension phase (up to interim data lock 10 August 2012) were considered. Pts (2–19 yrs) with active SJIA were enrolled and received CAN 4 mg/kg or placebo sc every 4 weeks for 12 weeks. CAN naïve pts who entered the trials and received at least one dose of CAN were included in this analysis (N=178 CAN naïve pts). Descriptive efficacy analyses of adapted ACR-JIA responses at Week 12 are provided for the BN and BE pts groups.

## Results

At baseline, there were 66 (37%) BN pts whereas anakinra (ANA), tocilizumab (TCZ), etanercept (ETN) and adalimumab (ADA), were the biologics received by 78 (44%), 10 (6%), 58 (33 %) and 9 (5%) pts, respectively. The main reasons for discontinuation of biologics in BE group (n=112) was lack of efficacy (ANA, n=32; TCZ, n=7; ETN, n=56; ADA, n=9) or safety/tolerability (ANA, n=20; TCZ, n=4, ETN, n=0). At Week 12, the BN and BE groups were similar in aACR-JIA 30 and 50 response rates (Week 2: aACR-JIA 30: 80% vs 80%; aACR-JIA 50: 76% vs 67%; Week 12: aACR-JIA 30: 76% vs 67%; aACR-JIA 50: 74% vs 65%). Numerically higher aACR-JIA 70 and 90 response rates were achieved in BN vs. BE pts ( Week 2: aACR-JIA 70: 67% vs 52%; aACR-JIA 90: 36% vs 37%; Week 12: aACR-JIA 70: 70% vs 55%; aACR-JIA 90: 61% vs 42%). aACR-JIA 70 and 90 response rates were similar in pts previously exposed to ANA vs those not exposed to ANA at 12 weeks (aACR-JIA70: 58% vs.63%; aACR-JIA 90:47% vs 50% ). Compared to pts who discontinued ANA due to lack of efficacy, there was a trend towards higher aACR-JIA 70 and 90 response rates at Week 12 in pts who stopped ANA for other reasons (aACR-JIA70: 34% vs.74%; aACR-JIA90: 25% vs. 63%). A higher aACR-JIA 30, 50, 70 and 90 response rates were observed in TCZ naïve pts vs. those pts exposed to TCZ (n=10) [aACR-JIA30: 71% vs.50%; aACR-JIA50: 70% vs. 50%; aACR-JIA70: 61% vs.50%; aACR-JIA90: 49% vs. 40%]. Higher aACR-JIA 70 and 90 responses were observed for ETN naïve pts vs. those exposed to ETN [aACR-JIA70: 67% vs. 48%; aACR-JIA90: 58% vs. 31%]; while ADA- naïve pts had similar responses to CAN as ADA-exposed pt (aACR-JIA 70: 61% vs 56%) and they had higher aACR-JIA 90 response (aACR-JIA90: 50% vs. 22%).

## Conclusion

In general, pts previously exposed to biologics achieved aACR-JIA 50,70 and 90 responses to CAN quickly in the first 2 weeks, and maintained their response up to Week 12; albeit at a numerically lower level than biologic-naïve pts. These data support the consistent efficacy of CAN across different subgroups of pts.

## Disclosure of interest

P. Quartier Grant / Research Support from: Abbvie, BMS, Chugai-Roche, Novartis, Pfizer and SOBI, Consultant for: Abbvie, Chugai-Roche, Novartis, Pfizer, Servier and SOBI, Speaker Bureau of: Chugai-Roche, MEDIMMUNE, Novartis, Pfizer, A. Grom Consultant for: Novartis, Roche, NovImmune, N. Ruperto Grant / Research Support from: To Gaslini Hospital: Abbott, Astrazeneca, BMS, Centocor Research & Development, Eli Lilly and Company, "Francesco Angelini", Glaxo Smith & Kline, Italfarmaco, Novartis, Pfizer Inc., Roche, Sanofi Aventis, Schwarz Biosciences GmbH, Xoma, Wyeth Pharmaceuticals Inc., Speaker Bureau of: Astrazeneca, Bristol Myers and Squibb, Janssen Biologics B.V.,Roche, Wyeth/Pfizer, H. Brunner Consultant for: Novartis, Genentech, Pfizer, UCB, AstraZeneca, Biogen, Boehringer-Ingelheim, Regeneron, Paid Instructor for: Novartis, Speaker Bureau of: Novartis, Genentech, K. Schikler Grant / Research Support from: Pfizer, Novartis, Abbvie, Roche, Genentech, Forest, Speaker Bureau of: Abbvie, Novartis, M. Erguven: None declared., L. Goffin Consultant for: Novartis, Pfizer , M. Hofer Grant / Research Support from: Novartis, Pfizer, Abbvie, T. Kallinich Grant / Research Support from: Novartis, Speaker Bureau of: Roche, Novartis, ALK, K. Marzan Grant / Research Support from: Novartis, C. Gaillez Employee of: Novartis, K. Lheritier Shareholder of: Novartis, Employee of: Novartis, K. Abrams Shareholder of: Novartis, Employee of: Novartis, A. Martini Grant / Research Support from: The Gaslini Hospital, which is the public Hospital where I work as full time employee, has received contributions to support the PRINTO research activities from the following companies: Bristol Myers and Squibb, Centocor Research & Development, Glaxo Smith & Kline,Novartis,Pfizer Inc, Roche, Sanofi Aventis, Schwarz Biosciences GmbH , Speaker Bureau of: Abbott, Bristol MyersSquibb, Astellas, Behringer, Italfarmaco, MedImmune, Novartis, NovoNordisk, Pfizer,Sanofi,Roche, Servier, D. Lovell Grant / Research Support from: National Institutes of Health- NIAMS , Consultant for: Astra-Zeneca, Centocor, Amgen, Bristol Meyers Squibb, Abbott, Pfizer, Regeneron, Roche, Novartis, UBC, Forest Research Institute, Horizon, Johnson & Johnson, Speaker Bureau of: Novartis, Roche
